# The mitochondrial genome of *Formicosepsis* sp. (Diptera: Cypselosomatidae)

**DOI:** 10.1080/23802359.2019.1623103

**Published:** 2019-07-10

**Authors:** Xin Li, Liang Wang, Zhu Li, Peng Hou, Ding Yang

**Affiliations:** a College of Plant Protection, China Agricultural University, Beijing, China;; b Beijing Museum of Natural History, Beijing, China

**Keywords:** Cypselosomatidae, phylogeny, mitochondrial genome, Formicosepsis

## Abstract

The mitochondrial genome of *Formicosepsis* sp. was sequenced and annotated as the first representative of family Cypselosomatidae and superfamily Nerioidea. The part mitochondrial genome of *Formicosepsis* sp. is 15,144 bp totally, consisting of 13 protein-coding genes, two rRNAs, and 22 transfer RNAs, which has a similar gene structure with other published species of Diptera. The nucleotide composition biases toward A and T is 76.4% of the entirety. IQ-tree analysis revealed that the outgroups *Anopheles oryzalimnetes* and *Simulium variegatum* diverged from the rest. The Nerioidea represented by *Formicosepsis* sp. was sister group to Opomyzoidea. The Sciomyzoidea consisting of Sciomyzidae and Sepsidae is a monophyletic clade at the base of Acalyptratae.

The Cypselosomatidae is a small family with body size from minute to medium (2.0–3.7 mm). These brown to black and yellow species is distributed in all biogeographic regions (McAlpine 1987; McAlpine 1966; Carvalho-Filho and Esposito 2011). The family, which is placed basally within the Nerioidea (Carvalho-Filho and Esposito 2011), were characterized by head with 1 reclinate and 2 proclinate fronto-orbital setae together with large, divergent postvertical setae and costa without strong setae, not broken near humeral crossvein, broken at end of subcostal or not distinctly so, terminating at end of vein M; subcostal not terminating independently in costa; vein R_4 + 5_ and M converging apically (McAlpine 1997).

Specimens of *Formicosepsis* sp. were collected in Xishuangbanna National Nature Reserve, Mengla, Yunnan, China (E101.562809, N21.494403) and identified by Xin Li and Ding Yang. The specimens were deposited in the Entomological Museum of China Agricultural University, Beijing.

The genomic DNA was extracted from adult’s whole body using the DNeasy DNA Extraction kit (TIANGEN) and stored at −20 °C refrigerator. The mito-genome of *Formicosepsis* sp. contains 22 transfer RNA genes, 13 protein-coding genes, and two ribosomal RNA genes, which were close to other Diptera flies reported before (Zhao et al. 2013; Kang et al. 2016; Li et al. 2017; Zhou et al. 2017; Yang et al. 2019). The mito-genome nucleotide composition of *Formicosepsis* sp. was 39.9% of A, 36.5% of T, 9.2% of G, 14.4% of C, and A + T content is 76.4%. All PCGs started with codon ATG and terminated with TAA.

There are 11 species retrieved from NCBI and 1 new sequenced data in phylogeny analysis, the genbank accession numbers are list following: *Anopheles oryzalimnetes* NC_030715, *Simulium variegatum* NC_033348, *Nemopoda mamaevi* NC_026866, *Trypetoptera punctulata* MK644823, *Ceratitis capitata* NC_000857, *Cestrotus liui* NC_034922, *Spaniocelyphus pilosus* NC_034924, *Drosophila melanogaster* NC_024511, *Drosophila yakuba* NC_001322, *Formicosepsis sp. MK644824, *Liriomyza bryoniae* NC_016713, and *Liriomyza trifolii* NC_014283. Thirteen protein-coding genes (PCGs) were used to reconstruct phylogeny relationship with IQ-tree (Schmidt et al. 2015; Hoang et al. 2018). The topology was given and bootstrap support numbers are shown in Figure 1. IQ-tree analysis revealed that the outgroups *Anopheles oryzalimnetes* and *Simulium variegatum* diverged from the rest. The Nerioidea represented by *Formicosepsis* sp. was sister group to Opomyzoidea. Nerioidea and Opomyzoidea were nested in Ephydroidea while Lauxanioidea and Tephritoidea were assigned to be sister groups. The Sciomyzoidea was monophyletic at the base of Acalyptratae.

**Figure 1.**
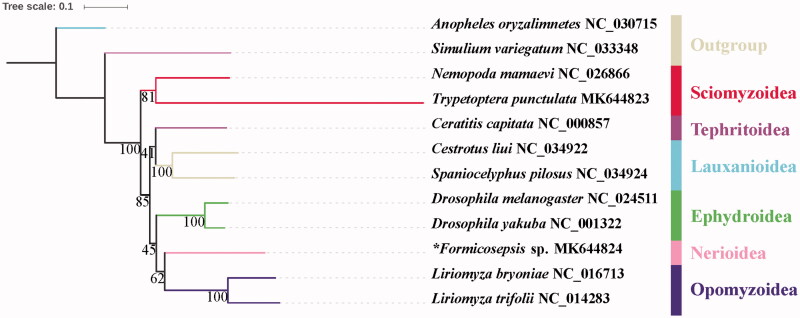
The phylogenetic tree of IQ-tree analysis based on 13PCGs; “*” indicated new sequenced data in this study.
